# A Kinematic Study of Prosodic Structure in Articulatory and Manual Gestures: Results from a Novel Method of Data Collection

**DOI:** 10.5334/labphon.75

**Published:** 2017-03-13

**Authors:** Jelena Krivokapić, Mark K. Tiede, Martha E. Tyrone

**Affiliations:** 1 University of Michigan, US; 2 Haskins Laboratories, US; 3 Long Island University Brooklyn, US

**Keywords:** Prosodic boundaries, prosodic prominence, speech production, gestures, electro- magnetic articulometry, EMA, motion capture, Vicon

## Abstract

The primary goal of this work is to examine prosodic structure as expressed concurrently through articulatory and manual gestures. Specifically, we investigated the effects of phrase-level prominence (Experiment 1) and of prosodic boundaries (Experiments 2 and 3) on the kinematic properties of oral constriction and manual gestures. The hypothesis guiding this work is that prosodic structure will be similarly expressed in both modalities. To test this, we have developed a novel method of data collection that simultaneously records speech audio, vocal tract gestures (using electromagnetic articulometry) and manual gestures (using motion capture). This method allows us, for the first time, to investigate kinematic properties of body movement and vocal tract gestures simultaneously, which in turn allows us to examine the relationship between speech and body gestures with great precision. A second goal of the paper is thus to establish the validity of this method. Results from two speakers show that manual and oral gestures lengthen under prominence and at prosodic boundaries, indicating that the effects of prosodic structure extend beyond the vocal tract to include body movement.^1^

## 1. Introduction

The term prosody refers to the suprasegmental structure of the utterance, which encodes prominence and phrasal organization (e.g., Ladd, 2001; Jun, 2005; Byrd & Saltzman, 2003). In speech, it is expressed through tonal and temporal properties, and, as recent studies have begun to show, through body gestures (e.g., McNeill et al., 2001; Mendoza-Denton & Jannedy, 2011 for English; Krahmer & Swerts, 2007 for Dutch). The goal of this paper is to examine the multimodal expression of prosodic structure by investigating how the temporal properties of both articulatory and manual gestures are affected by prosodic phrasing and phrase-level prominence.

Languages differ in how they mark prominence prosodically, in terms of whether phrasal prominence is indicated by culminative marking of the head of a prosodic unit (e.g., by pitch accent or pitch range expansion), by phrasing, or by both (Jun, 2005; Ladd, 1996/2008). If marked culminatively, languages further differ regarding what the phonetic correlates of prominence are (e.g., Beckman, 1986; Jun, 2005; Ladd, 1996/2008). In English, phrase level prominence is signaled by marking words with pitch accents, which highlight important or new information and serve rhythmic purposes. A prosodic phrase in English contains at least one pitch accent (the nuclear pitch accent, which is by definition the last pitch accent in a phrase) and optionally additional ones, and the pitch accent is anchored on the stressed syllable of the word. Furthermore, words can receive focus (specifically narrow, broad, and contrastive) or be deaccented (see Table 1 for examples), and the phonetic correlates of prominence can vary depending on the focus structure (Breen et al., 2010 for English; Mücke & Grice, 2014 for German).^2^ In English, in addition to tonal properties, phrase-level prominence is marked by temporal properties (e.g., Breen et al., 2010), which are the focus of this study. A number of studies have examined unaccented compared to accented syllables and have found that acoustic segments and articulatory gestures in accented syllables are longer than in unaccented syllables (e.g., Turk & Sawusch, 1997; Turk & White, 1999; Edwards et al., 1991; Cho, 2006; de Jong et al., 1993; Fowler, 1995 for English), although such studies have primarily compared unaccented words to accented words under contrastive focus. Studies specifically examining duration in focus find in general that focus lengthens the duration of a word or syllable. Thus in English, contrastively-focused words are acoustically longer than deaccented words (Cooper et al., 1985), and narrow and broad focus lead to acoustic lengthening as well (Eady et al., 1986). A recent study of German by Mücke and Grice (2014) was the first to examine the effect of the different degrees of prominence under investigation in our study, namely, deaccented, broad, narrow, and contrastive focus. In their acoustic analysis, they found that deaccented and broad-focused stressed syllables are shorter than narrow-focused stressed syllables, which in turn are shorter than contrastive-focused stressed syllables. This suggests that prominence lengthening might be cumulative, increasing from deaccented through broad, narrow, and contrastive focus. However, the kinematic comparisons (duration, maximum displacement, peak velocity, and time-to-peak velocity of the lip opening movement of the onset consonant of the stressed syllable) showed limited and speaker-specific evidence of such cumulative increase in focus-related lengthening. In a related study, Cho and Keating (2009) examined the effects of lexical (primary vs. secondary stress) and phrasal prominence (accented vs. unaccented) and found no evidence of cumulative lengthening.

Prosodic phrasing functions to group words together into chunks that are used by speakers and listeners for language processing. Prosodic phrases are hierarchically organized, with higher units dominating lower units (e.g., Selkirk, 1984; Nespor & Vogel, 1986). The number of prosodic phrase levels is somewhat controversial (cf. Ladd, 1996/2008; Gordon, 2016), but for English, at least a minor and a major phrase above the word level are typically assumed (Shattuck-Hufnagel & Turk, 1996). These will be referred to here as the intermediate phrase (ip) and the Intonation Phrase (IP), respectively, following Beckman and Pierrehumbert’s 1986 approach. Prosodic boundaries (the boundaries between prosodic phrases) have specific tonal and temporal properties. Boundaries are marked by tones, which may lead to rising, falling, or level pitch (in Beckman & Pierrehumbert’s 1986 model, these are the phrase accents and boundary tones marking ip and IP boundaries, respectively). The temporal properties of boundaries are referred to as boundary-adjacent lengthening. At boundaries, articulatory gestures become temporally longer both phrase-initially and phrase-finally (e.g., Edwards et al., 1991; Fougeron & Keating, 1997; Byrd & Saltzman, 1998; Byrd, 2000; Cho, 2006 for English). In the acoustic signal, this is reflected in lengthening of boundary-adjacent segments, i.e., phrase-final and phrase-initial lengthening (e.g., Oller, 1973; Wightman et al., 1992; Shattuck-Hufnagel & Turk, 1998; Cho 2006 for English), and pauses can optionally occur at strong prosodic boundaries (see overview in Krivokapić 2007b; for the articulatory properties of pauses, see Ramanarayanan et al., 2010, 2013 for English, and Katsika, 2016 for Greek). The temporal effects increase with boundary strength, such that there is more articulatory and acoustic lengthening at hierarchically higher boundaries (e.g., Wightman et al., 1992; Byrd & Saltzman, 1998; Shattuck-Hufnagel & Turk, 1998; Byrd, 2000; Cho, 2006 for English). The lengthening effects of the boundary are local, i.e., they do not occur far away from the boundary, and the effect is strongest close to the boundary and decreases further away from it (e.g., Byrd et al., 2006; Krivokapić, 2007a for English). These properties are accounted for by the π-gesture model (Byrd & Saltzman, 2003). Within this model, prosodic boundaries are clock-slowing gestures (π-gestures). The π-gestures extend over an interval, and as the result of the activation of the π-gesture, co-active speech gestures– gestures under the scope of the π-gesture–become temporally longer and less overlapped. Hierarchically higher prosodic boundaries have a stronger activation of the π-gesture, which leads to stronger boundary effects. Furthermore, due to the shape of the π-gesture (gradually increasing to a maximum activation and then gradually decreasing in activation), the effect of the boundaries is strongest at the boundary and decreases with distance from it. Thus, the model predicts that at boundaries gestures become temporally longer, that the lengthening is local (affecting the gestures co-active with the π-gesture), and that the effects decrease with distance from the boundary and increase with boundary strength.

Based on numerous and broad-ranging studies in linguistics, psychology, and anthropology, it is well-established that speech and body gestures are inter-related, and moreover, that body gestures are an essential component of spoken language interaction (e.g., Kendon, 2004; McNeill, 1985, 1992, 2005 for general studies; Kendon, 1972; Özyürek et al., 2007; for Dutch, Willems et al., 2007 for English). Studies by Kendon (1972, 2004) and by McNeill et al. (2001) have demonstrated that body gestures help structure discourse. More recently, studies have focused on the relationship between manual gestures (either finger-tapping or pointing) and prominence (see Wagner et al., 2014, for an overview), and to a lesser extent on eyebrow movement and head nods in prominence (e.g., Hadar et al., 1983 for English; Krahmer & Swerts, 2007 for Dutch; Munhall et al., 2004 for Japanese; Beskow et al., 2006; Al Moubayed & Beskow, 2009 for Swedish). There is evidence that body gestures are timed to prominent syllables (e.g., de Ruiter, 1998; Swerts & Krahmer, 2010 for Dutch; McNeill, 1992 general study; Loehr, 2004; Mendoza-Denton & Jannedy, 2011 for English; Rochet-Capellan et al., 2008 for Brazilian Portuguese; Esteve-Gibert & Prieto, 2013 for Catalan). Specifically, recent studies have indicated that it is the gesture apex (i.e., the displacement maximum of a gesture) that is timed to the prominent syllable (e.g., Leonard & Cummins, 2011 for English; Esteve-Gibert & Prieto, 2013 for Catalan). While a number of questions related to this coordination remain open (see Esteve-Gibert & Prieto, 2013, Wagner et al., 2014; Krivokapić, 2014), this work provides strong evidence of a close link between speech and body gestures.

Research on the relationship between body gestures and prosodic boundaries is limited, but there is support for the idea that speech and body gestures act jointly to form a prosodic boundary. Early evidence that phrasing and discourse are indicated by body gestures was given in Kendon (1972) and in McNeill et al. (2001) for English. Barkhuysen et al. (2008) found indications that Dutch speakers tend to turn their eyes and head away phrase-medially and to return their gaze and head position toward an interlocutor phrase-finally. Speakers in their study also had more eye blinks and head nods phrase-finally than phrase-medially. Similarly, Ishi et al. (2014) found in a spontaneous speech study of Japanese that 80% of head nods occurred during the phrase-final syllable. Zeller et al. (2016) found in a corpus study of Swedish that when a speaker continues speaking after a discourse boundary, acoustic segments preceding the boundary are longer than for other types of discourse boundaries when the accompanying gesture phase is static (i.e., the hands are in a motionless phase of a gesture) but not when the accompanying gesture phase is dynamic.^3^ Finally, two studies on Catalan found that stress and boundary position have an effect on the timing of the gesture apex (i.e., a gesture’s displacement maximum) for pointing gestures (Esteve-Gibert & Prieto, 2013) and for head nods (Esteve-Gibert et al., 2014), thus showing that body gestures, similar to prominence, are affected by prosodic boundaries and are coordinated both with boundaries and with prominence. Together, these studies provide evidence of a relationship between body gestures and prosodic boundaries. However, this research is still in its beginning stages.

To fully understand the relationship between prosodic structure, body gestures, and speech, we will need to investigate not only the co-occurrence and exact coordination of speech and body gestures (i.e., which precise point in speech is coordinated to which precise point in the body gesture) and how the coordination is affected by prosodic structure, but also whether and how the durations of body gestures are affected by prosodic structure. One of the central manifestations of prosodic structure in speech is temporal lengthening of speech gestures under prominence and at prosodic boundaries (as discussed above). Thus, if body gestures are also organized under prosodic structure, they should exhibit lengthening as well. Only a few studies have examined this question for prominence. The evidence indicates that manual gestures co-vary with articulatory gestures. Specifically, in syllable repetition tasks, finger-tapping movements increase in amplitude and temporally lengthen when they co-occur with a prominent syllable, and increased finger movement amplitude leads to increased prominence in observed acoustic and/or articulatory signals (Kelso et al., 1983; Parrell et al., 2014 for English). Deictic gestures also temporally lengthen when co-occurring with contrastive prominence (Rusiewicz et al., 2014 for English), and acoustic segments lengthen when co-occurring with a body gesture (Krahmer & Swerts, 2007). Furthermore, listeners perceive words co-produced with body gestures as more prominent (Krahmer & Swerts, 2007). Lengthening of body gestures at prosodic boundaries has yet to be investigated. To the best of our knowledge, the only study examining this question is Krivokapić’s (2014), a small spontaneous speech study with acoustic and video data of English that found indication that body movements lengthen, such that at the ends of prosodic phrases there is lengthening of body movements starting during speech and ending during pauses. However, no evidence of lengthening of other body movements was found (e.g., there was no lengthening of body movements starting during the pause and ending during speech).

The goal of the current study is to examine how prosodic structure is manifested in manual gestures and specifically to determine whether there is evidence of prominence- and boundary-related lengthening. The language examined is American English. Three experiments are presented. Experiment 1 examines the effect of phrase-level prominence on speech and manual gestures. Experiments 2 and 3 test the effects of prosodic boundaries on speech and manual gestures (Experiment 2 examines phrase-initial, and Experiment 3 phrase-final lengthening). Overall, the prediction is that if the effects of prosodic structure extend beyond the vocal tract, manual gestures will show similar temporal properties as gestures of the vocal tract. Specifically, for Experiment 1, based on research on the temporal manifestations of prosodic prominence discussed above, we predict that:

1 Manual and vocal tract gestures will display lengthening under prominence.
2 The lengthening will be cumulative (increasing with degree of prominence), along a range from deaccented (least) through broad, narrow, and contrastive focus (most).

Based on the research on prosodic boundaries in speech discussed above and on the π-gesture model of Byrd and Saltzman (2003), for Experiments 2 and 3, we predict that:

3 Manual and vocal tract gestures will lengthen at prosodic boundaries.
4 Lengthening will be local to the boundary.
5 Lengthening will be cumulative (increasing with boundary strength).

Experiments 2 and 3 are a first step towards establishing whether prosodic boundaries have a comparable lengthening effect across the two modalities of spoken language communication. Experiment 1 is the first study to examine prominence lengthening kinematically in both speech and manual gestures produced concurrently (rather than, for example, in the kinematics of finger-tapping or in the acoustic signal for speech, as has been done previously) and to examine the effects of different types of prominence on lengthening in manual gestures. The results of these studies will help address questions of whether and how speech and body gestures are integrated to form prosodic structure.

Part of the difficulty in addressing the relationship between speech and body gestures is that comparable and precisely measured kinematic data are needed for both. Most previous studies have used video and acoustic data to investigate this relationship, but such data do not allow for a direct comparison of gestural timing across manual and vocal tract gestures. A few studies have used kinematic data for speech and for manual gestures, but could only examine external articulations (e.g., lip aperture in Rochet-Capellan et al., 2008, using motion capture), thus not allowing for the examination of most consonants and vowels, or they could acquire only a very limited range of manual movement (e.g., finger-tapping on the opposite shoulder in Parrell et al., 2014, using electromagnetic articulometry). While it could be argued that lengthening–which is the focus of this study–can be investigated from acoustic and video data, examination of lengthening is more accurate with kinematic data (e.g., the exact onset and end of speech gestures during pauses can be accurately determined) and the data can also be more reliably analyzed (e.g., the onset and apex of body gestures are difficult to determine from video data; see discussion in Seyfeddinipur, 2006). Our new approach involves recording speech and body movement concurrently, using electromagnetic articulometry (EMA) and a motion capture system (Vicon). This is, to the best of our knowledge, the first time that the kinematics of vocal tract gestures and body movements have been recorded simultaneously, and thus one of the goals of our study is to establish the feasibility of this data collection method.

## 2. Methods

The three experiments presented here were carried out together. Since the methods are similar across experiments, we will begin by presenting the stimuli for each experiment, then discuss the general data collection and analysis procedures.

### 2.1. Stimuli

#### 2.1.1. Stimuli Experiment 1

Experiment 1 examines lengthening under phrase-level prominence, specifically in four focus conditions: deaccented, broad focus, narrow focus, and contrastive focus. The stimulus utterance was “Anna wants to see Bob. In the morning if possible”, presented as the answer in a question-answer pairing. The questions were designed to elicit the four different types of focus (the questions are given in Table 1; see Mücke & Grice, 2014 for a similar procedure). The target word in each utterance was *Bob* (thus the syllable was always stressed), and the constrictions of interest were the two /b/ consonants. The sentence following the target word served to ensure that the boundary produced was an IP boundary rather than possibly a discourse boundary, since less is known about these in speech production, and to ensure that the phrase-final articulation of the target word is more controlled by the post-boundary phrase. Participants were asked to read the question (displayed on a computer monitor) silently and the answer aloud, and to point with the index finger of their dominant hand to a picture (also on the monitor) representing *Bob* while saying the target word. The utterances were produced twelve times, resulting in a total of 48 utterances (4 prominence types *x* 12 repetitions). They were pseudo-randomized in blocks of four utterances.

#### 2.1.2. Stimuli Experiment 2

Experiment 2 examines phrase-initial lengthening. Three utterances varying the type of boundary (word boundary, ip–intermediate phrase boundary, IP–Intonation Phrase boundary) were constructed. The boundary-adjacent target word was a neologism (the name *MIma* or *miMA*, with stress on the first or on the second syllable, indicated by capitalization).^4^ Twelve repetitions of each utterance were read from a computer screen for a total of 72 productions (3 boundaries *x* 2 target words *x* 12 repetitions). The utterances were pseudo-randomized in blocks of six. Table 2 shows the stimuli for the target word *MIma*. The utterances with the second target word were identical except that the target word was *miMA*. The examined boundary is before the target word and the constriction of interest is the first /m/ in each target word. Participants were asked to point to the appropriate picture of a doll (named either *miMA* or *MIma*) while saying the target word.

#### 2.1.3. Stimuli Experiment 3

Experiment 3 examines phrase-final lengthening. The stimuli consisted of three utterances varying the type of boundary (word boundary, ip boundary, IP boundary). The utterances contained neologisms as target words (the names *DIdad* or *diDAD*, with the stress on the first and on the second syllable respectively).^5^ Different target words were used than in the second experiment so that the target word would end in a consonant, since consonants are typically easier to label than vowels. The relevant boundary is after the target word, and the relevant constriction is the final /d/. There were twelve repetitions of each utterance for a total of 72 productions (3 boundaries *x* 2 target words *x* 12 repetitions). The utterances were pseudo-randomized in blocks of six. Table 3 shows the stimuli for the target word *DIdad*. The utterances with the target word *diDAD* were the same except for the target word. As in the previous experiments, participants read the utterances from a computer screen and were asked to point to the appropriate picture of a doll (named *DIdad* or *diDAD*) while saying the target word.

### 2.2. Participants

The data for the three experiments were collected as part of a larger study not discussed here. The participants were 2 native speakers of American English, 1 male (age 27) and 1 female (age 23), with no known self-reported speech or hearing deficits. They were paid for their participation and naïve as to the purpose of the study.

It should be noted that one of the main interests of this study is in the novelty of the combined Vicon/EMA method–outlined in the following section–for examining manual gestures and speech articulation concurrently. Thus, the findings from applying this method to the 2 speakers are intended to be illustrative and are not generalizable at this stage. It is also difficult at this stage to assess how many speakers are necessary for achieving an appropriately powerful analysis of the data presented in this study.

### 2.3. Data collection

**Figure 1:**
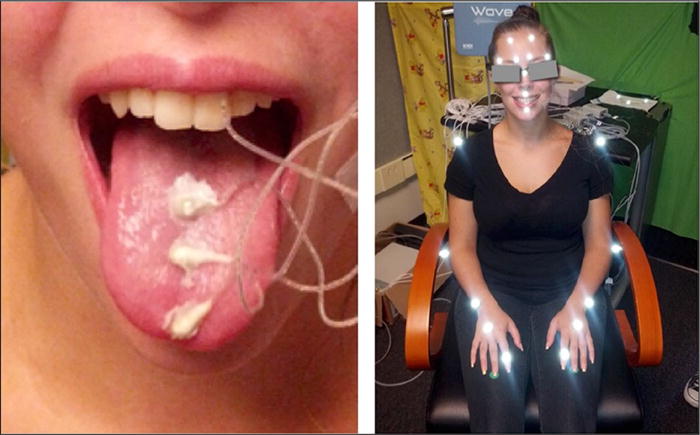
Movement tracking. EMA sensors on the left and motion capture markers on the right.

The audio signal, gestures of the vocal tract, and body movements were recorded concurrently. Vocal tract gestures were recorded using a 5D electromagnetic articulometer system (EMA; WAVE, Northern Digital), at a sampling rate of 100 Hz (Berry, 2011). These data were collected synchronously with the audio signal, which was sampled at 22025 Hz. Four EMA sensors were placed midsagitally on the tongue tip, tongue body, and tongue dorsum (see Figure 1), and on the lower incisors (to track jaw movement). Three additional sensors were placed on the upper incisors and on the left and right mastoid processes as references to correct for head movement. Body movement was acquired separately using a motion capture system (Vicon; Oxford, UK) which employed six infrared-sensitive cameras and a visible-light camera to track the 3D positions of reflective markers synchronized with video, both at a sampling rate of 100 Hz (Tyrone et al., 2010). Nineteen motion capture markers were placed on the forehead and nose (for reference alignment with EMA), adjacent to the lips and eyebrows, and on the arms and hands, including one marker on each index finger (Figure 1). In post-processing, data from the concurrently recorded audio, EMA, Vicon, and video streams were temporally aligned through cross-correlation of head movement reference data, and trajectories of head-mounted sensors and markers were converted to a coordinate system centered on the upper incisors and aligned with the speaker’s occlusal plane. Although the EMA sensors were coupled using wires taped to facial skin and the Vicon markers require line-of-sight to the tracking cameras, this data collection method nonetheless allows for relatively unconstrained movement of the head and arms, essential for body gestures and to support a natural conversational setting.

**Figure 2:**
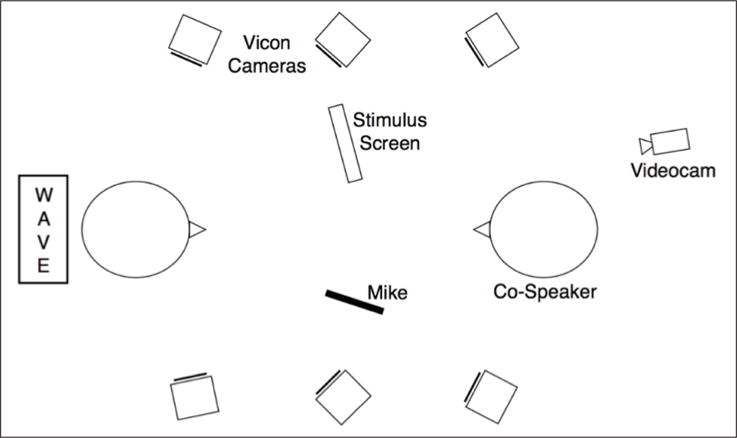
Experimental setup.

The experimental setup is shown in Figure 2. The EMA transmitter unit was placed at the back of the participant’s head. The Vicon cameras were positioned as shown in Figure 2, located high enough to have an unobstructed view of the reflective markers. Participants had a clear view of the monitor on which stimuli were presented. During the experiment, participants read the utterances as they appeared on the monitor and pointed, with the index finger of their dominant hand, to a picture of a doll (also on the screen), while reading the associated target word. The finger was resting on a green paper dot, which was attached close to the participant’s knee (where their hand rested naturally), following a similar procedure in de Ruiter (1998) and Rochet-Capellan et al. (2008). The experimenter monitored each production and asked participants to repeat incorrectly produced utterances. The day before each experiment, there was a practice session during which the participants were introduced to the procedure and practiced the production of the novel words and stimuli utterances.

### 2.4. Prosodic verification

The utterances were screened for appropriate prosodic production. The different types of prosodic prominence that are the focus of Experiment 1 are difficult to objectively transcribe since they can be expressed with various types of pitch accents. Three labelers (the first author and two transcribers naïve to the purposes of the experiment but with linguistic training and experience in prosody) listened to each utterance to ascertain whether the speakers produced the targeted focus structure. The criterion was whether a particular utterance could be the answer to the associated context question. Utterances on which not all transcribers agreed were excluded from the analysis. Speaker 1 produced all words targeted to be deaccented with some degree of prominence (mostly with narrow focus) and these utterances were excluded. It is likely that for this speaker, it was difficult to produce a deaccented noun while producing a deictic gesture at the same time (cf. Krahmer & Swerts, 2007, who found that producing a gesture leads to an increase in duration of the associated speech). Apart from that, all utterances were produced with the appropriate focus structure, except for two utterances which Speaker 1 did not produce as targeted (one with targeted broad and one with targeted narrow focus). These two utterances were excluded as well.

Boundaries were labeled using the Tone and Break Indices labeling system (Beckman & Ayers Elam, 1997), and it was checked whether the boundaries were produced in the targeted location and with the targeted strength, and whether ip and IP boundaries were produced at other, unexpected locations. In both experiments, the targeted ip and IP boundaries were realized as IP boundaries. In the remainder of the paper they will be therefore referred to as IP1 (for the targeted ip condition) and IP2 (for the targeted IP condition). As expected from the stimuli utterances, boundaries were also produced at all commas, at periods, and nowhere else. In addition, a number of word boundaries were realized as strong prosodic boundaries, and some target words were mispronounced or sentences produced with a disfluency; these sentences were excluded, as shown in Table 4. Note that for Speaker 2, only seven word boundaries (out of 24) in Experiment 2 were produced as word boundaries (the remaining 17 were produced with IP boundaries) and this condition was therefore excluded entirely for this participant.

## 3. Data analysis and results: Experiment 1

### 3.1. Labeling: Experiment 1

**Figure 3:**
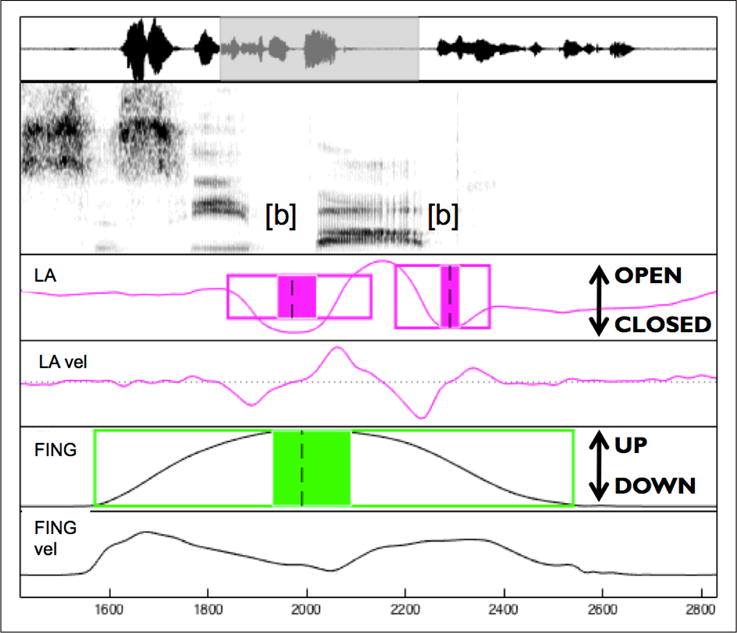
Labeling example for *Bob*. The identified landmarks shown here are (for /b/): gesture onset (left edge of the box), nucleus onset (left edge of the shaded box), maximum constriction (dashed line), nucleus offset (right end of the shaded box), gesture offset (right end of the box). LA: lip aperture trajectory and velocity, FING: finger vertical displacement trajectory and tangential velocity.

Articulatory gestures were labeled using a semi-automatic procedure (mview; Haskins Laboratories). The constrictions that are expected to show most of the effect of prominence are the two consonants and the vowel; since the vowel and consonant constrictions overlap in articulation, we expect that the full effect of prominence is going to be seen on the consonant constrictions (see also Figure 3, where the consonant gestures occur in part during the acoustic signal of the vowel). We therefore examine the initial and the final /b/ (C1 and C2 respectively) in the target word *Bob*. The closing and the opening movements of these constrictions were analyzed on the lip aperture (LA) signal determined by the Euclidean distance between lip markers. We also labeled the manual (pointing) gesture, identified from movements of a marker attached to the index finger (distal phalanx) of the dominant hand (right hand for Speaker 1 and left hand for Speaker 2). Here we refer to the movement towards the picture as the pointing movement and the movement returning to the resting position (the green dot) as the return movement. The temporal landmarks were identified using velocity criteria. For these gestures, the identified landmarks used in this study were (see also Figure 3): gesture onset (20% of onset peak velocity), peak velocity of the closing/pointing movement, maximum constriction/finger displacement (velocity minimum), peak velocity of the opening/return movement, gesture offset (20% of offset peak velocity). We used these landmarks to calculate the variables of interest for speech and pointing gestures. For C1 and C2 and for the manual gesture we calculated the values described in Table 5 below.

Note that C1 OPENDUR and OPENDURACC, and C2 CLOSEDUR and CLOSEDURACC are the movements occurring during the vowel, and are expected to show the strongest effects of prominence. Previous research has found that the effects of prominence decrease further away from the prominence center (Bombien et al., 2013 for German). While this is not the focus of our study, we include these movements (C1 CLOSEDUR and CLOSEDURACC and C2 OPENDUR and OPENDURACC) to test for evidence of decrease of strength of effect as well. We cannot examine this kind of lengthening effect decrease in the manual gesture without first understanding more precisely the coordination of speech and manual gestures, given that it is not clear where to expect the lengthening effect to be strongest.

### 3.2. Statistical analysis: Experiment 1

A one-way ANOVA tested the effect of prominence with the levels broad, narrow, and contrastive for Speaker 1, and the levels deaccented, broad, narrow, and contrastive for Speaker 2, on the dependent variables (CLOSEDUR, CLOSEDURACC, OPENDUR, OPENDURACC for C1 and C2, and POINTINGDUR, POINTINGDURACC, RETURNDUR, RETURNDURACC for the manual gesture). In cases where an ANOVA was significant (*p* < 0.05), a post-hoc test (Fisher’s PLSD) was conducted to test the effect of the different levels of prominence on the dependent variables.

### 3.3. Results: Experiment 1

The results for the two speakers are given in Tables 6 and 7. Both speakers exhibit evidence of lengthening under prominence for both speech and manual gestures. For the speech gestures for Speaker 1, there is lengthening on both opening and closing movements for both consonants, although not all examined variables show lengthening (there is no effect of prominence for C1 CLOSEDURACC and for C2 CLOSEDURACC and C2 OPENDUR). This speaker overall distinguishes two degrees of prominence, such that, in general, under contrastive focus gestures are longer than under broad and narrow focus. Broad and narrow focus showed different effects in C2 CLOSEDUR and C2 OPENDURACC with broad being longer than narrow for the former variable, and the reverse was true for the latter variable. Lengthening did not decrease with distance from the syllable center, in that there were two degrees of lengthening distinguished throughout the speech gestures. For the manual gesture there is evidence of lengthening on one movement, namely on RETURNDUR, where, in parallel to the speech gestures, contrastive focus is longer than broad and narrow focus. Surprisingly, there was no effect on the pointing movement for either of the two examined variables. To examine whether there might be evidence of lengthening on a portion of the manual gesture, we further examined the duration of the PLATEAUX (the duration of the shaded box in Figure 3, from nucleus onset to offset) of the manual gestures (the nucleus onset is 20% of the local peak velocity during deceleration into the point of maximum constriction; the nucleus offset is 20% of the local peak velocity during acceleration away from the point of maximum constriction). A one-way ANOVA on the PLATEAUX found no effect of prominence. A further possibility is that the manual gesture is coordinated with speech in such a way that the pointing movement precedes the prominence to a large extent, so that the effect of prominence would only occur on the return movement. To examine this, the lag between C1 and the pointing movement was examined. Specifically, the lag from the onset of the pointing movement to the onset of C1 was calculated. If most of the pointing movement precedes the onset of the C1 gesture, it could be argued that the pointing movement is not under the scope of prominence. The argument is that since the C1 closing movement shows effects of prominence, we know that the effect of prominence starts at least with C1, but might not start early enough to cover the pointing movement if the pointing movement precedes speech. The results, however, indicate that this is not the case. The average lag (with standard deviation) between C1 onset and pointing movement onset (pointing movement onset subtracted from C1 onset) is –34.5 (53.9) ms for broad, –38.2 (50.56) ms for narrow, and –38.5 (58.85) ms for contrastive focus, indicating that the pointing movement starts a bit after the C1 consonant gesture.

For Speaker 2, for the speech gesture there is evidence of prominence-related lengthening on the C1 closing and opening movements and on the C2 closing movement. Thus the overall scope of lengthening is shorter than for Speaker 1, as it does not extend to the opening movement of C2. There is no effect of prominence on the C1 CLOSEDURACC, or on the C2 CLOSEDURACC. Speaker 2 distinguishes up to four degrees of prominence lengthening, starting with two degrees for the C1 CLOSEDUR movement, increasing to three for the C1 OPENDUR and to four for C1 OPENDURACC, and decreasing again to two degrees for the C2 CLOSEDUR movement. This provides some evidence of the strongest effect being at the syllable center. The cumulative increase in prominence is as predicted: Contrastive is longer than narrow, which is longer than broad, and the shortest is the deaccented level. For the manual gesture, there is again evidence of lengthening only on the returning part of the gesture, on both RETURNDUR and RETURNDURACC. As for Speaker 1, we therefore conducted a one-way ANOVA on the duration of the PLATEAUX (the duration from nucleus onset to offset) of the manual gestures. There was a significant effect, such that contrastive focus is longer than the other prominence levels. Thus the effect for this speaker is evident earlier in the gesture. The manual gesture overall distinguishes two degrees of prominence, with contrastive focus always longer than broad and deaccented focus and longer than all other prominence levels for PLATEAUX. Narrow focus is longer than broad and deaccented focus for the RETURNDUR movement and longer than broad focus for the RETURNDURACC. While manual gestures do not show the cumulative lengthening that was observed for speech gestures, a noticeable parallel between speech and manual movement is that the last speech movement (C2 CLOSEDUR) exhibits the same contrasts as the RETURNDUR of the manual gesture.

The lag between speech and manual gestures was also examined. For this speaker, the manual gesture onset precedes the C1 speech gesture on average (with standard deviation) by 170.6 (82.48) ms in the deaccented condition, 157.5 (83.44) ms in the broad focus condition, 118.5 (72.55) ms in the narrow, and 107.5 (44.33) ms in the contrastive condition. The pointing gesture duration (onset to maximum displacement) is 453.02 (67.18) ms for deaccented, 490.85 (72.42) ms for broad, 506.82 (87.75) ms for narrow, and 514.16 (83.6) ms for contrastive focus. Thus the beginning of the pointing movement precedes the onset of C1, and so it might be outside of the scope of prominence. Together with the findings for PLATEAUX, which showed lengthening, this indicates that while there might be lengthening over part of the pointing movement this might not lead to statistically significant lengthening of the entire pointing movement, since the beginning of the movement is outside of the scope of prominence.

## 4. Data analysis and results: Experiment 2

### 4.1. Labeling: Experiment 2

**Figure 4:**
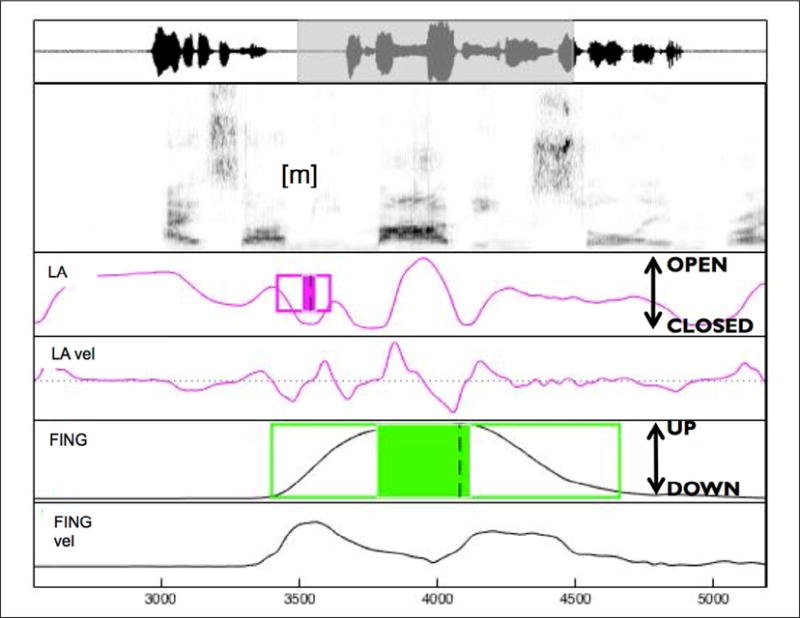
Labeling example for *miMA*, for the utterance, “There are other things. I saw miMA being stolen in broad daylight by a cop”. The identified landmarks shown here are (for /m/): gesture onset (left edge of the box), nucleus onset (left edge of the shaded box), maximum constriction (dashed line), nucleus offset (right end of the shaded box), gesture offset (right end of the box). LA: lip aperture trajectory and velocity. FING: finger vertical displacement trajectory and tangential velocity.

As in Experiment 1, articulatory gestures were labeled using a semi-automatic procedure (mview; Haskins Laboratories). The constriction of interest is the initial /m/ in the target words (*miMA* and *MIma*), which is the constriction closest to the boundary, and is expected to show the most lengthening (Byrd & Saltzman, 2003). The closing and opening movements of these constrictions were analyzed on the lip aperture (LA) signal. As in Experiment 1, we also labeled the manual gesture. The temporal landmarks used in this experiment were the same as in Experiment 1, namely gesture onset, peak velocity of the closing/pointing movement, maximum constriction/finger displacement, peak velocity of the opening/return movement, and gesture offset (see also Figure 4). We used these landmarks to calculate the same variables for speech and manual gestures as in Experiment 1 (CLOSEDUR, CLOSEDURACC, OPENDUR, OPENDURACC for lip aperture and POINTINGDUR, POINTINGDURACC, RETURNDUR, RETURNDURACC for the pointing gesture). Note that the closing/pointing movements (CLOSEDUR, CLOSEDURACC for lip aperture and POINTINGDUR, POINTINGDURACC for the manual gesture) are the movements closest to the boundary, where the effect of the boundary is expected to be the strongest, while the opening/return movements are further away from the boundary, and a weaker effect of the boundary is expected (Byrd & Saltzman, 2003).

### 4.2. Statistical analysis: Experiment 2

The examined durations were *z*-scored by articulator (finger or LA) and by target word, for each speaker separately, in order to focus on the boundary effects and remove the effects of stress. One-way ANOVAs tested the effect of the boundary (levels: word, IP1, and IP2 boundary) on the eight dependent variables (CLOSEDUR, CLOSEDURACC, OPENDUR, OPENDURACC for lip aperture and POINTINGDUR, POINTINGDURACC, RETURNDUR, RETURNDURACC for the manual gestures). In cases where an ANOVA was significant (*p*<0.05), a post-hoc analysis (Fisher’s PLSD) was conducted testing the effect of the different levels of boundary strength on the dependent variables.

While we are presenting the results of both target words pooled, two-way ANOVAs (factors: boundary *x* stress) gave essentially the same results as the one-way ANOVAs presented here: there was an effect of boundary, such that boundaries lead to lengthening, and for some cases an effect of stress. Speaker 2 showed a stress *x* boundary interaction for OPENDUR and OPENDURACC in that there was no effect of stress when the boundary was IP2, but for the word and IP1 boundary, stress on the first syllable led to longer duration than stress on the second syllable.

### 4.3. Results: Experiment 2

For Speaker 1 (see Table 8), a one-way ANOVA shows a main effect of prosodic boundaries on the LA closing and manual pointing durations, and post-hoc analysis (Fisher’s PLSD) shows that these movements are shorter in duration at word boundaries than at IP1 or IP2 boundaries. Further away from the boundary, for LA OPENDUR, a one-way ANOVA shows an effect of the boundary and Fisher’s PLSD shows again that gestures at word boundaries are shorter than gestures at IP1 or IP2 boundaries. There was no effect on the manual return movement duration or acceleration duration, which is unsurprising, since this movement is far away from the boundary. For Speaker 2 (see Table 9), the analysis showed that LA CLOSEDUR and CLOSEDURACC at word boundaries are shorter than at IP1 or IP2 boundaries. Further away from the boundary, for LA OPENDUR and LA OPENDURACC, movements at word and IP2 boundaries are shorter than at IP1 boundaries. For the manual gesture, POINTINGDUR shows a three-way distinction, such that movement at word boundaries is shorter than at IP1 boundaries, which is in turn shorter than at IP2 boundaries. POINTINGDURACC shows a two-way distinction, such that it is longer at the IP2 boundary than at the word boundary. There was no effect on the manual return movement duration or acceleration duration, which as explained above for Speaker 1, is unsurprising.

## 5. Data analysis and results: Experiment 3

### 5.1. Labeling: Experiment 3

**Figure 5:**
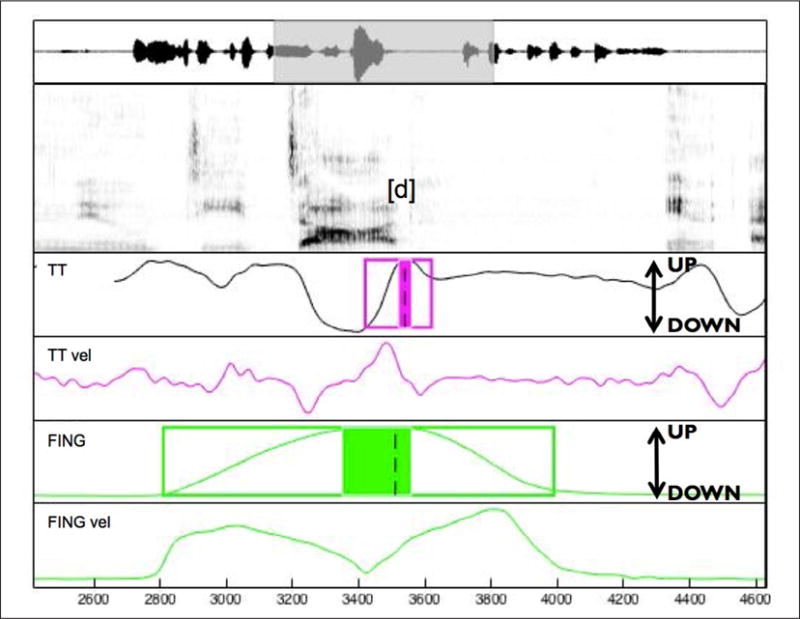
Labeling example for *diDAD*, for the utterance, “Mary would like to get the new diDAD. In Ette this would be quite easy”. The identified landmarks (for /d/) shown here are: gesture onset (left edge of the box), nucleus onset (left edge of the shaded box), maximum constriction (dashed line), nucleus offset (right end of the shaded box), gesture offset (right end of the box). TT: vertical tongue tip trajectory and vertical velocity. FING: finger vertical displacement and tangential velocity.

Data were labeled as in the previous experiments, and a sample token is shown in Figure 5. The gestures of interest in Experiment 3 are the final /d/ in the target words (*diDAD* and *DIdad*), which is the constriction closest to the boundary, and the manual pointing gesture. For /d/ the vertical tongue tip (TT) trajectory was tracked and temporal landmarks were identified using the vertical velocity signal. The temporal landmarks for the manual gesture were identified using tangential velocity measures, as in Experiments 1 and 2. Gesture onset and peak velocity of the closing and opening movement for /d/ were highly variable and could not be systematically identified across repetitions; we therefore analyzed only the opening movement (from maximum constriction to gesture release) for both manual and TT constriction. This is the movement closest to the boundary, where the effect of the boundary is expected to be the strongest. Given that the closing movement could not be examined, an additional boundary duration measure (BNDDUR) was taken, as a further way to estimate boundary strength. BNDDUR was defined as the TT movement from the pre-boundary maximum constriction to the onset of the post-boundary gesture /n/ (see, e.g., Byrd & Saltzman, 1998, for the use of trans-boundary intervals as a measure of boundary strength). This measure captures the opening movement duration of /d/ and any pause duration that the boundary might contain. Pause duration is also a good correlate of boundary strength, as studies have found that stronger boundaries tend to have longer pauses (see overview in Fletcher, 2010). Thus this measure assesses the boundary comprehensively and is a good indicator of boundary strength. The onset of the post-boundary constriction /n/ was identified using the same TT velocity criteria.

### 5.2. Statistical analysis: Experiment 3

The analyses for Experiment 3 were similar to those applied in Experiment 2. The durations were *z*-scored and one-way ANOVAs tested the effect of the boundary (levels: word boundary, IP1, and IP2) on the opening movement durations (OPENDUR) for the TT gesture, RETURNDUR for the manual gesture, and for the entire /d/:/n/ boundary duration (BNDDUR). In cases where the ANOVAs were significant (*p*<0.05), Fisher’s PLSD was conducted testing the effect of the different levels of boundary strength on the dependent variables. For Speaker 2, who did not produce the word boundary, a one-way ANOVA was conducted testing the effect of boundary (levels IP1 and IP2 boundary) on the OPENDUR, BNDDUR, and the manual RETURNDUR.

As in Experiment 2, we present the results of both target words pooled, since we are not interested in the effect of stress. A two-way ANOVA (boundary *x* stress) shows essentially the same results as for the pooled target words: While there is an effect of boundary in the direction that the pooled target words show, and in one instance there is an effect of stress, there is no stress *x* boundary interaction.

### 5.3. Results: Experiment 3

The results for both speakers are given in Table 10. For Speaker 1, a one-way ANOVA showed a main effect of boundary on TT OPENDUR, manual RETURNDUR, and BNDDUR. Fisher’s PLSD showed that the duration of the TT opening movement was longer in IP1 than in word and IP2 boundaries. Similarly, the manual RETURNDUR was longer in IP1 than in IP2 boundaries. For the boundary duration there is a three-way distinction with IP2 boundaries having longer durations than IP1 boundaries, which in turn are longer in duration than word boundaries. For the second speaker, there is no effect of boundary on either TT or manual gesture, but there is an effect on BNDDUR, such that the IP2 boundary is longer than the IP1 boundary.

## 6. Discussion and Conclusion

Using a novel method of data collection, this study examined whether manual gestures, like speech gestures, exhibit boundary- and prominence-related lengthening. We have shown that electromagnetic articulometry and motion capture methods can be used together for simultaneous collection of vocal tract gestures, acoustic data, video, and body movement. This approach will allow researchers to examine the kinematic properties of speech and body gestures and the coordination of these gestures with far greater precision than has been possible so far.

Prosodic effects were seen in both speech and manual gestures. We start with prominence effects in speech. Speaker 2 showed cumulative lengthening, with up to four degrees of prominence strength distinguished. The increase in lengthening is in the predicted direction: Contrastive > narrow > broad > deaccented. While such cumulative lengthening is a known prosodic boundary effect, it has not been previously shown for prominence for English (but see Mücke & Grice, 2014 for German). Another similarity to known boundary effects is that there is evidence for the effect of prominence decreasing with distance from the center of the syllable for Speaker 2.

For both speakers, manual gestures show a parallel to speech gestures, in that both types of gestures show lengthening. For Speaker 1, both speech and manual gestures show two degrees of lengthening with contrastive focus being longer than narrow or broad focus. For Speaker 2, while there is no cumulative lengthening in the manual gesture, the effect on the RETURNDUR is exactly parallel (i.e., the same four comparisons are significant, as can be seen in Table 7) to the last speech movement that shows an effect (C2 CLOSEDUR). Overall, the fact that the manual gesture shows lengthening comparable to what occurs in the speech gestures is evidence of the tight integration of speech and body movement in prominence.

The fact that the pointing movement of the manual gesture did not show lengthening for Speaker 1 was surprising, although Parrell et al. (2014) found something similar. In their study, the lengthening effect on the finger-tapping movement occurred after the stress-related lengthening of the speech gestures. For Speaker 2, there was no evidence of lengthening of the pointing movement either, but this result might be due to the coordination of speech and manual gestures, in that the beginning of the manual gesture preceded the onset of C1, thus possibly being out of the scope of prominence lengthening. Another possibility might be that the lengthening of the return movement is related to the upcoming prosodic boundary. This is not a likely scenario since all sentences had a strong boundary, but to test for this possibility, we further examined pause durations. If there were an effect of focus type on pause duration, the return movement lengthening could be related to this effect. However, there was no effect of focus type on pause duration for either speaker, therefore this explanation has to be excluded. An interesting follow-up study might be to examine whether there is any effect of cognitive load on the delayed effect of prominence on the manual gesture. It could be that the prominence task was more demanding than the boundary task, given that in the prominence study speakers had to produce the same string of words with different prosodic structures, and thus had to pay quite a bit of attention to the contextualizing question. This increased cognitive load could lead to a destabilizing of the timing between speech and body gestures. Alternatively, the constrained and somewhat artificial nature of the elicitation task might have led to a corresponding constraint on manual gesture production, i.e., it might have restricted the scope for timing variation.

With respect to prosodic boundaries, it was found that in both modalities movements closest to the boundary (the TT opening movement and manual return movement phrase-finally, and the LA closing movement and manual pointing movement phrase-initially) lengthen. These are the movements which typically display the most prominent boundary effects (Byrd & Saltzman, 2003). Phrase-finally, only these movements were examined. Phrase-initially, further away from the boundary, there is evidence of lengthening for the speech gestures for both speakers and no effect for the manual gesture for either speaker. The lack of lengthening on the return movement is unsurprising, given that the manual movements are much longer in duration. In other words, phrase-initially the manual return movement is beyond the expected scope of the boundary (see, e.g., Byrd et al., 2006) and therefore not predicted to lengthen.

Turning to phrase-initial lengthening in more detail, Speaker 1 shows a strong parallel between speech and manual gestures, with both modalities distinguishing two boundary strengths, namely IP2 and IP1 boundaries being longer than word boundaries. The results for Speaker 2 also show comparable boundary effects. For this speaker, two boundaries are distinguished in the LA closing movements (CLOSEDUR and CLOSEDURACC) and up to three degrees of boundary strength are distinguished in the manual movement (IP2 > IP1 > word for POINTINGDUR, and IP2 > word for POINTINGDURACC). This crucially shows that more than one boundary strength can be distinguished in manual gestures, as evidenced for speech in previous studies (e.g., Wightman et al., 1992; Byrd & Saltzman, 1998; Cho, 2006).

Phrase-finally, the parallel between speech and manual gestures is also strong, with one speaker showing two boundary strengths in both modalities and one speaker not showing the effects of boundary on either manual or TT movements (although there was a temporal effect of the boundary, as evidenced in the measure of the boundary duration BNDDUR).

Overall, the effect of the boundary was similar across the two modalities. Thus, at boundaries–at least in our small study–manual gestures exhibit properties typical of speech gestures, namely, manual gestures lengthen, the lengthening is local, and it increases with boundary strength.

Our study thus provides evidence of parallel behavior in speech and manual gestures, as shown in the lengthening at boundaries and under prominence, and in the cumulative lengthening of the manual gesture for boundaries. The question then arises regarding the source of the prosodic effects on manual gestures. One possibility is that they emerge through the π-gesture. In the π-gesture model, boundaries are gestures which locally (at boundaries) slow the utterance clock. Their effect is strongest at the boundary and decreases with distance from it. It has been suggested previously that this model might also account for prominence-related lengthening (Byrd & Saltzman, 2003; Saltzman et al., 2008; Bombien et al., 2010, 2013). It has also been suggested that this model could account for prominence effects in body gestures (Parrell et al., 2014), given that the π-gesture is expected to affect any movement that is controlled by the same clock as speech. Given the tight relationship between speech and body gestures, it is likely that they are controlled by the same clock. The similar behavior of speech and manual gestures in our study provides further support for this idea.

To conclude, in a fine-grained study of prominence-related lengthening, we have provided evidence that manual gestures lengthen under prominence. While prominence-related lengthening effects have been previously shown for finger-tapping and speech gestures, and for pointing gestures and acoustic measures of speech, this is the first time that a detailed analysis of lengthening of both speech and manual gesture has been conducted. The boundary studies are a first step towards examining the kinematic properties of manual gestures at prosodic boundaries and provide initial evidence that body gestures exhibit boundary lengthening. If body gestures did not show lengthening, the claim that prosodic control extends beyond the vocal tract would be difficult to maintain. These results thus add crucial evidence to the body of work showing prosodic properties of body gestures. Future research is needed to verify these findings from a larger sample of speakers, and to address how body gestures and speech are integrated into one system. The π-gesture model might give rise to the prosodic effects in both domains, but the observed differences in how the effect is manifested (specifically the prominence effects for Speaker 1 and possibly Speaker 2) still need to be explained.

## Figures and Tables

**Table 1:** Question-answer pairs for Experiment 1. The target word is *Bob* and the constrictions of interest are the two /b/ consonants.

Condition	Context question	Answer
*deaccented*	Does Lenny want to see Bob?	Anna [wants to see Bob]_deaccented_. In the morning if possible.
*broad*	What is going on?v	[Anna wants to see Bob]_broad_. In the morning if possible.
*narrow*	Who does Anna want to see?	Anna wants to see [Bob]_narrow_. In the morning if possible.
*contrastive*	Does Anna want to see Mary?	Anna wants to see [Bob]_contrastive_. In the morning if possible.

**Table 2:** Stimuli for Experiment 2 for the target word *MIma*. The target boundary is before *MIma*, and the relevant constriction is the first /m/ (underlined here but not when the stimuli were presented to participants).

Condition	Utterance
1. word	There are other things. I saw MIma being stolen in broad daylight by a cop.
2. ip	Mary would like to see Shaw, MIma, Beebee, and Ann while she is here.
3. IP	There are other things I saw. MIma being stolen was the most surprising one.

**Table 3:** Stimuli for Experiment 3 for the target word *DIdad*. The target boundary is after *DIdad*, and the relevant constriction is the last /d/ (underlined here but not in the experiment).

Condition	Utterance
1. word	Mary would like to get the new DIdad in Ette this year for her birthday.
2. ip	Mary would like to get the new DIdad, Ynette, and Bobby for her birthday.
3. IP	Mary would like to get the new DIdad. In Ette this would be quite easy.

**Table 4:** Number of tokens excluded from the analysis for Experiments 2 and 3.

Boundary	Experiment 1 (phrase initial)		Experiment 2 (phrase final)	

	Speaker 1	Speaker 2	Speaker 1	Speaker 2
word	4 excluded (IP boundary produced)	8 excluded (IP boundary produced)	0	17 sentences produced with IP boundary (condition excluded)
Ip/IP1	2 excluded (incorrect target word/disfluency)	1 (incorrect target word)	0	0
IP/IP2	3 excluded (incorrect target word/disfluency)	1 (disfluency)	0	0

**Table 5:** Examined variables.

Speech variable	Description	Pointing gesture variable	Description
CLOSEDUR	duration of the constriction closing movement (from onset to maximum constriction)	POINTINGDUR	duration of the pointing movement (from onset to maximum finger displacement)
CLOSEDURACC	constriction closing movement acceleration duration (from onset to peak velocity)	POINTINGDURACC	pointing movement accelera- tion duration (from onset to peak velocity)
OPENDUR	duration of the constriction opening movement (from maximum constriction to gesture release)	RETURNDUR	duration of the return movement (from maximum finger displacement to gesture release)
OPENDURACC	constriction opening movement acceleration duration (from maximum constriction to peak velocity)	RETURNDURACC	return movement acceleration duration (from maximum finger displacement to peak velocity)

**Table 6:** Results for prominence lengthening, Speaker 1. Means (SE) in ms., ANOVA, Fisher’s PLSD.

C1 LA closing movement duration (CLOSEDUR)	
broad = 86.36 (3.4)	contrastive, broad: *p* < 0.0001
narrow = 92.73 (3.4)	contrastive, narrow: *p* < 0.0001
contrastive = 116.67 (3.25)	contrastive > broad, narrow
*F*(2,31) = 23.3485, *p* < 0.0001	
**C1 LA opening movement duration (OPENDUR)**	
broad = 134.55 (5.31)	contrastive, broad: *p* = 0.0052
narrow = 145.46 (5.31)	contrastive > broad
contrastive = 156.67 (5.08)	
*F*(2,31) = 4.5335, *p* = 0.0187	
**C1 LA opening movement acceleration duration (OPENDURACC)**	
broad = 75.45 (4.51)	contrastive, broad: *p* = 0.0027
narrow = 81.82 (4.51)	contrastive, narrow: *p* = 0.0320
contrastive = 95.83 (4.31)	contrastive > broad, narrow
*F*(2,31) = 5.6358, *p* = 0.0082	
**C2 LA closing movement duration (CLOSEDUR)**	
broad = 117.5 (3.99)	broad, narrow: *p* = 0.0139
narrow = 102.5 (3.99)	broad > narrow
contrastive = 111.82 (3.41)	
*F*(2,24) = 3.6096, *p* = 0.0426	
**C2 LA opening movement acceleration duration (OPENDURACC)**	
broad = 37.47 (3.88)	contrastive, broad: *p* = 0.0003
narrow = 53.2 (3.63)	narrow, broad: *p* = 0.0071
contrastive = 58.51 (3.1)	contrastive, narrow > broad
*F*(2,23) = 9.1662 *p* = 0.0012	
**Manual return movement duration (RETURNDUR)**	
broad = 515.65 (10.64)	contrastive, broad: *p* = 0.0042
narrow = 525.91 (10.64)	contrastive, narrow: *p* = 0.0228
contrastive = 561.2 (10.19)	contrastive > broad, narrow
*F*(2,31) = 5.3263, *p* = 0.0103	

**Table 7:** Results for prominence lengthening, Speaker 2. Means (SE) in ms., ANOVA, Fisher’s PLSD.

C1 LA closing movement duration (CLOSEDUR)	
deaccented = 151.57 (10.7)	contrastive, deaccented: *p* = 0.0073
broad = 167.5 (10.7)	narrow, deaccented: *p* = 0.0111
narrow = 191.67 (10.7)	contrastive, narrow > deaccented
contrastive = 194.17 (10.7)	
*F*(3,44) = 3.6245, *p =* 0.0201	
**C1 LA opening movement duration (OPENDUR)**	
deaccented = 167.5 (9.4)	contrastive, deaccented: *p* < 0.0001
broad = 190 (9.4)	contrastive, broad: *p* = 0.0001
narrow = 230.83 (9.4)	narrow, deaccented: p = 0.0001
contrastive = 262.5 (9.4)	narrow, broad: *p* = 0.0037
*F*(3,44) = 20.2095, *p* < 0.0001	contrastive, narrow: *p* = 0.0217
	contrastive > narrow > deaccented, broad
**C1 LA opening movement acceleration duration (OPENDURACC)**	
deaccented = 111.67 (8.57)	contrastive, deaccented: *p* = 0.0001
broad = 138.33 (8.57)	contrastive, broad: *p* = 0.0001
narrow = 166.67 (8.57)	narrow, deaccented: *p* = 0.0001
contrastive = 198.33 (8.57)	contrastive, narrow: *p* = 0.0122
*F*(3,44) = 18.9008, *p* < 0.0001	narrow, broad: *p* = 0.0240
	broad, deaccented: *p* = 0.0331
	contrastive > narrow > broad > deaccented
**C2 LA closing movement duration (CLOSEDUR)**	
deaccented = 110 (2.88)	contrastive, broad: *p* = 0.0001
broad = 105 (2.88)	contrastive, deaccented: *p* = 0.0037
narrow = 123.33 (2.88)	narrow, broad: *p* = 0.0001
contrastive = 122.5 (2.88)	narrow, deaccented: *p* = 0.0021
*F*(3,44) = 10.0398, *p* < 0.0001	contrastive, narrow > broad, deaccented
**Manual return movement duration (RETURNDUR)**	
deaccented = 512.5 (21.02)	contrastive, broad: *p* = 0.0001
broad = 483.65 (21.02)	contrastive, deaccented: *p* = 0.0011
narrow = 574.88 (21.02)	narrow, broad: *p* = 0.0037
contrastive = 615.95 (21.02)	narrow, deaccented: *p* = 0.0417
*F*(3,44) = 8.0968, *p* = 0.0002	contrastive, narrow > broad, deaccented
**Manual return movement acceleration duration (RETURNDURACC)**	
deaccented = 297.5 (27.59)	contrastive, broad: *p* = 0.0001
broad = 265 (27.59)	contrastive, deaccented: *p* = 0.0013
narrow = 353.62 (27.59)	narrow, broad: *p* = 0.0281
contrastive = 431.67 (27.59)	contrastive > broad, deaccented
*F*(3,44) = 6.9983, *p* = 0.0006	narrow > broad
**Manual PLATEAUX**	
deaccented = 170 (21.46)	contrastive, deaccented: *p* = 0.0033
broad = 172.5 (21.46)	contrastive, broad: *p* = 0.0042
narrow = 179.17 (21.46)	contrastive, narrow: *p* = 0.0076
contrastive = 264.17 (21.46)	contrastive > narrow, broad, deaccented
*F*(3,44) = 4.4559, *p* = 0.0081	

**Table 8:** Results for phrase-initial lengthening, Speaker 1. Means (SE) in *z*-scores, ANOVA, Fisher’s PLSD.

LA closing movement duration (CLOSEDUR)	
Word = –0.61 (0.2)	Word, IP1: *p* < 0.0001
IP1 = 0.56 (0.19)	Word, IP2: *p* = 0.0194
IP2 = 0.05 (0.19)	IP2, IP1 > Word
*F*(2,60) = 9.2728, *p* = 0.0003	
**LA opening movement duration (OPENDUR)**	
Word = –0.45 (0.21)	Word, IP1: *p* = 0.0222
IP1 = 0.25 (0.2)	Word, IP2: *p* = 0.0422
IP2 = 0.18 (0.21)	IP2, IP1 > Word
*F*(2,60) = 3.2620, *p* = 0.0452	
**Manual pointing movement duration (POINTINGDUR)**	
Word = –0.51 (0.20)	Word, IP1: *p* = 0.0443
IP1 = 0.07 (0.19)	Word, IP2: *p* = 0.0060
IP2 = 0.31 (0.20)	IP2, IP1 > Word
*F*(2,60) = 4.2851, *p* = 0.0182	

**Table 9:** Results for phrase-initial lengthening, Speaker 2. Means (SE) in *z*-scores, ANOVA, Fisher’s PLSD.

LA closing movement duration (CLOSEDUR)	
Word = –0.97 (0.2)	Word, IP1: *p* < 0.0001
IP1 = 0.38 (0.17)	Word, IP2: *p* < 0.0001
IP2 = 0.2 (0.17)	IP2, IP1 > Word
*F*(2,59) *=* 15.0459, *p* < 0.0001	
**LA closing movement acceleration duration (CLOSEDURACC)**	
Word = –0.6 (0.23)	Word, IP1: *p* = 0.0277
IP1 = 0.09 (0.19)	Word, IP2: *p* = 0.0032
IP2 = 0.33 (0.19)	IP2, IP1 > Word
*F*(2,59) *=* 4.8671, *p =* 0.0111	
**LA opening movement duration (OPENDUR)**	
Word = –0.61 (0.19)	Word, IP1: *p* < 0.0001
IP1 = 0.46 (0.15)	IP1, IP2: *p* = 0.0035
IP2 = –0.21 (0.15)	IP1 > IP2, Word
*F*(2,59) *=* 10.5402, *p =* 0.0001	
**LA opening movement acceleration duration (OPENDURACC)**	
Word = –0.66 (0.21)	Word, IP1: *p* < 0.0001
IP1 = 0.68 (0.17)	Word, IP2: *p* = 0.006
IP2 = –0.22 (0.17)	IP1 > IP2, Word
*F*(2,59) *=* 13.4276, *p* < 0.0001	
**Manual pointing movement duration (POINTINGDUR)**	
Word = –0.79 (0.2)	Word, IP1: *p* = 0.0120
IP1 = –0.11 (0.17)	Word, IP2: *p* < 0.0001
IP2 = 0.48 (0.17)	IP2, IP1: *p* = 0.0150
*F*(2,59) = 11.8749, *p* < 0.0001	IP2 > IP1 > Word
**Manual pointing movement acceleration duration (POINTINGDURACC)**	
Word = –0.51 (0.24)	Word, IP2: *p* = 0.0108
IP1 = 0.046 (0.2)	IP2 > Word
IP2 = 0.31 (0.2)	
*F*(2,59) = 3.5046, *p* = 0.0365	

**Table 10:** Results for phrase-final lengthening, both speakers. Means (SE) in *z*-scores, ANOVA, Fisher’s PLSD.

TT opening movement duration OPENDUR (Speaker 1)	
Word = –0.3 (0.19)	Word, IP1: *p* = 0.0111
IP1 = 0.42 (0.19)	IP1, IP2: *p* = 0.0499
IP2 = –0.13 (0.19)	IP1 > Word, IP2
*F*(2,69) *=* 3.7224, *p =* 0.0292	
**Manual return movement duration RETURNDUR (Speaker 1)**	
Word = 0.01 (0.19)	IP1, IP2: *p* = .0003
IP1 = 0.5 (0.19)	IP1 > IP2
IP2 = –0.51 (0.19)	
*F*(2,69) = 7.2415, *p =* 0.0014	
**Boundary duration BNDDUR (Speaker 1)**	
Word = –1.17 (0.09)	Word, IP1: *p* < 0.0001
IP1 = 0.14 (0.09)	Word, IP2: *p* < 0.0001
IP2 = 0.96 (0.09)	IP1, IP2: *p* < 0.0001
*F*(2,69) = 154.4370, *p* < 0.0001	IP2 > IP1 > Word
**Boundary duration BNDDUR (Speaker 2)**	
IP1 = –0.91 (0.11)	
IP2 = 0.77 (0.1)	IP2 > IP1
*F*(1,46) = 129.3240, *p* < 0.0001	

## Footnotes

2 The literature provides a variety of terms to describe whether the information in a sentence is newly introduced to the discourse or already present. We are interested in the kinematic properties of prominence as expressed in speech and manual gestures, and therefore, following Mücke and Grice (2014), we chose fine-grained focus distinctions. For a discussion of deaccenting, broad and narrow focus, see Ladd (1980 Ladd (2008), and for narrow and contrastive focus, see Wagner (2012).

3 A gesture can be divided into gesture phases, namely a preparation movement, pre-stroke hold, stroke, post-stroke hold, and retraction/recovery movement (Kendon, 1972; Kita, 1993). The holds are the static phases of the gesture. The stroke (the only obligatory part of the gesture) is typically dynamic but does not have to be, and other movement phases of the gesture are always dynamic.

4 The stress manipulation was done as part of a separate study and will not be further discussed here.

5 As in Experiment 2, the stress manipulation was done as part of a separate study and will not be further discussed here.
